# Supplementary material for: Identification of photoperiod-induced specific miRNAs in the adrenal glands of Sunite sheep (Ovis aries)
Source: Front Vet Sci. 2022 Jul 22;9:888207. doi: 10.3389/fvets.2022.888207 (PMC9354845; doi:10.3389/fvets.2022.888207)
Supplement: Supplementary file 1 [file Data_Sheet_1.ZIP › Supplementary file/miRDeep2 file.zip!/miRDeep2 file/Novel_Structure/SP42A1/Novel_110.pdf]

Provisional ID	:	NC_019458.2_735
Score total	:	41.7
Score for star read(s)	:	3.9
Score for read counts	:	36.3
Score for mfe	:	0
Score for randfold	:	1.6
Score for cons. seed	:	
Total read count	:	83
Mature read count	:	82
Loop read count	:	0
Star read count	:	1

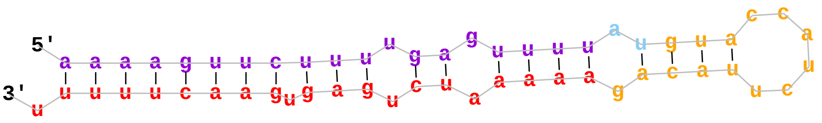

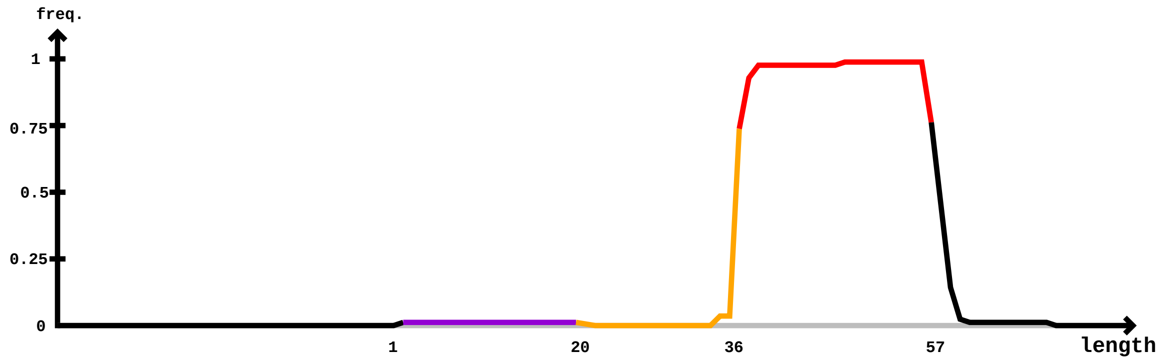


```
                                  Star                                    Mature
5'- auauuuuuauuuguaacuaaguaugggcugaaaaaaaaguucuuuugaguuuuauguaccaucuuacagaaaaaucugagugaacuuuuuggccagcccaguauuaaaua   -3'    obs
    auauuuuuauuuguaacuaaguaugggcugaaaaaaaaguucuuuugaguuuuauguaccaucuuacagaaaaaucugagugaacuuuuuggccagcccaguauuaaaua          exp
    .................(((...(((((((...((((((((((((.((.((((.((((......)))).)))).)).))).)))))))))...)))))))...)))....   reads     mm     sample
    ..................................aaaaguucGuuugaguuuu.........................................................   1         1      seq
    ...................................................................agaaaaaucugaAugaacuuuu.....................   1         1      seq
    ...................................................................agaaaaaucugaAugaacuuuuu....................   2         1      seq
    .....................................................................aaaaaucugaAugaacuuuu.....................   17        1      seq
    .....................................................................aaaaaucugaAugaacuuuuu....................   42        1      seq
    ......................................................................aaaaucugagugaacuuuu.....................   1         0      seq
    ......................................................................aaaaucugagugaacuuuuu....................   4         0      seq
    ......................................................................aaaaCcugagugaacuuuuu....................   1         1      seq
    ......................................................................aaaaucugagugaacuuuuug...................   1         0      seq
    ......................................................................aaaaucugagugaacuuuuugg..................   8         0      seq
    ......................................................................aaaaCcugagugaacuuuuugg..................   1         1      seq
    .......................................................................aaaucugagugaacuuuuu....................   2         0      seq
    .......................................................................aaaucugagugaacuuuuugg..................   1         0      seq
    .......................................................................aaaCcugagugaacuuuuuggc.................   1         1      seq
    ................................................................................ugaacuuuuuggccaCcccagu........   1         1      seq
```
